# Evaluation of neutrophil-to-lymphocyte ratio and platelet-to-lymphocyte ratio as potential markers for ulcerative colitis: a retrospective study

**DOI:** 10.1186/s12876-022-02571-9

**Published:** 2022-11-24

**Authors:** Wan Feng, Yajun Liu, Lei Zhu, Luzhou Xu, Hong Shen

**Affiliations:** Department of Gastroenterology, Affiliated Hospital of Nanjing University of Chinese Medicine (Jiangsu Province Hospital of Chinese Medicine), Nanjing, China

**Keywords:** Neutrophil-to-lymphocyte ratio, Platelet-to-lymphocyte ratio, Ulcerative colitis

## Abstract

**Purpose:**

Ulcerative colitis (UC) is a chronic idiopathic inflammatory disorder affecting the large intestine. Inflammatory biomarkers in UC are nonspecific, simple and cheap biomarker is needed. Our study aimed to explore the possible relationship of platelet-to-lymphocyte ratio (PLR) and neutrophil-to-lymphocyte ratio (NLR) with the disease activity in UC. Furthermore, the correlation of PLR or NLR with other clinical indicators was evaluated.

**Methods:**

We retrospectively reviewed the clinical data of UC patients presented to the Affiliated Hospital of Nanjing TCM University. A total of 306 UC patients were included in the study. Clinical characteristics, NLR, PLR, C-reactive protein (CRP), erythrocyte sedimentation rate (ESR), fecal calprotectin (FC) and other data were collected.

**Results:**

PLR and NLR were elevated in active UC patients than those in remission. The receiver-operating characteristic (ROC) analysis revealed the optimal cutoff of NLR for active UC was 2.19, with sensitivity and specificity of 78.8 and 65%, respectively. For PLR, the best cut-off value was 147.96, with sensitivity and specificity of 58.3 and 75%, respectively. Both NLR and PLR were positively correlated with CRP, ESR and FC.

**Conclusions:**

PLR and NLR were elevated in patients with active UC as compared with patients in remission. NLR and PLR could be used in patients with UC as noninvasive markers of disease activity.

## Introduction

Ulcerative colitis (UC) is a chronic idiopathic inflammatory disease affecting the large intestine. It is characterized by relapsing mucosal inflammation. The aim of treatment is to induce and maintain disease remission. Assessing disease activity may help in optimizing the management of UC patients. C-reactive protein (CRP), erythrocyte sedimentation rate (ESR) and fecal calprotectin (FC) are frequently used for activity assessment of UC. These markers are nonspecific and may be influenced by other causes of inflammation. Some patients with severely active UC may have a normal CRP or ESR [1].

Recently, hematological parameters neutrophil-to-lymphocyte ratio (NLR), and platelet-to-lymphocyte ratio (PLR) have been reported as inflammation indicators. They are helpful in assessing severity of many chronic diseases, such as chronic obstructive pulmonary disease, hepatic echinococcosis, rheumatoid arthritis and many inflammatory diseases [2–8]. Growing evidence also suggested that increased NLR or PLR indicates poor prognosis and/or survival for multiple cancers. It is reported that elevated pre-treatment NLR is related to shorter overall survival and progression free survival [9, 10]. Several studies have indicated that the level of NLR and PLR was elevated in inflammatory bowel disease (IBD) [11–16].

NLR and PLR can be easily derived from the complete blood count, which is simple and cheap. Several studies indicated NLR and PLR could be used to evaluate UC severity. However, these results are mainly based on a relatively small number of patients. These studies did not explore the correlation between NLR, PLR and FC. FC can be used as a non-invasive stool biomarker, we estimate the association between PLR or NLR with FC in this study. We aimed to compare the NLR and PLR of patients with UC during remission and active phase. Furthermore, the correlation of PLR or NLR with other clinical indicators was examined.

## Methods

### Study population

We retrospectively reviewed the clinical data of UC patients presented to the Affiliated Hospital of Nanjing TCM University from May 2017 to June 2021. We included hospitalized adult patients whose clinical data including general information, laboratory parameters (platelet count, neutrophil, and lymphocyte and calculation of NLR, PLR) could be accessed. Diagnosis was made using symptoms, endoscopic assessment, histology, and the absence of alternative diagnoses. Demographic and clinical data was extracted, including age, gender, neutrophil count, lymphocyte count, platelet count, ESR, CRP and FC.

### Clinical disease activity

Clinical disease activity was evaluated by Truelove and Witts criteria. The Truelove and Witts criteria is one of the most commonly used disease activity index based on number of bloody bowel movements per day, heart rate, hemoglobin level, ESR and temperature. As is described by previous studies [15, 17, 18], patients with moderate or severe UC were defined as active disease, while patients in the mild group were defined as remission. The disease extent was classified based on the Montreal Classification.

### Statistics

Continuous variables were expressed as median and interquartile range (IQR), and categorical variables were expressed as percentage. Mann-Whitney-U and Kruskal-Wallis test were used to determine the differences between groups. Correlations between PLR and NLR with clinical indicators were analyzed using the Spearman’s correlation coefficient. A receiver-operating characteristic (ROC) curve was constructed to differentiate active from inactive UC. A *P*-value < 0.05 was regarded as statistically significant. The data was statistically analyzed using SPSS 25.0 statistical software.

## Results

### Characteristics of participants

The study subjects consisted of 306 Chinese patients with UC. There were 169 males and 137 females. The median age was 46 years (interquartile range [IQR] 34–57). The median disease duration was 4 years. There were 156 patients with clinically active UC and 150 patients in remission. Of the 306 patients, the proportion of proctitis, left-sided colitis and pancolitis was 19.3, 34.3 and 46.4%, respectively. Use of 5-aminoasalicylates, steroids, and biologic agents was reported at 82.7, 9.8, and 6.5%, respectively (Table 1).

**Table 1:** Demographic and Clinical Characteristics of UC patients

Variables	median(IQR), n	%
Age (years)	46(34 -57)	
Gender		
men	169	55.2%
women	137	44.8%
Duration of disease(months)	48(12-84)	
Activity		
active	156	51%
remission	150	49%
Extent of disease		
Proctitis (E1)	59	19.3%
Left sided (E2)	105	34.3%
Pancolitis (E3)	142	46.4%
Treatment (%)		
5-ASA	253	82.7%
Steroids	30	9.8%
Immunomodulators	6	2%
Biologics	20	6.5%

*5-ASA* 5-aminosalicylates, *IQR* Inter-quartile range

Compared with patients in remission, neutrophil count and platelet count were elevated in the clinically active UC patients. FC, ESR and CRP were significantly higher in the active UC patients than those in remission. No differences in lymphocyte count were observed between the active and remission group (Table 2).

**Table 2:** Comparison of parameters in active and remission UC patients

	Active	Remission	*P* value
Age (years)	46 (32.5-58)	47.5 (35-56)	0.394
Sex (men/women)	97/59	72/78	0.013
Extent of disease			
Proctitis (E1)	14	45	
Left sided (E2)	49	56	
Pancolitis (E3)	93	49	
Neutrophil	5.16(3.83-6.59)	3.03(2.28-4.02)	<0.001
lymphocyte	1.6(1.3-2.18)	1.6(1.3-1.99)	0.509
Platelet count	266(203.2-336.2)	203.5(153.75-241)	<0.001
NLR	3 (2.22-4.49)	1.83(1.41-2.51)	<0.001
PLR	161.98(116.87-222.25)	122 (96.78-147.92)	<0.001
CRP	7.97(2.68-21.6)	1.84(1.32-3.12)	<0.001
ESR	22(10-38)	5(5-13)	<0.001
FC	976.55(647.38-1332.5)	89.75(42-567.75)	<0.001

Values are expressed as number (%), *IQR* Inter-quartile range, *UC* Ulcerative colitis, *ESR* Erythrocyte sedimentation rate, *CRP* C-reactive protein, *NLR* Neutrophil to lymphocyte ratio, *PLR* Platelet to lymphocyte ratio, *FC* Fecal calprotectin

### PLR and NLR were increased in active UC patients

The median NLR in patients with active and remission UC was 3 (IQR 2.22–4.49) and 1.83 (IQR 1.41–2.51), respectively (*p* < 0.001). The median PLR value was 161.98 (IQR 116.87–222.25) in clinically active UC patients in contrast to 122 (IQR 96.78–147.92) in remission phase (*p* < 0.001). PLR and NLR levels significantly elevated in patients with active disease than those found during the remission phase (Table 2).

Among UC patients, the majority were taking mesalazine. We excluded patients taking steroids and immunosuppressant, which may influence the leukocyte count. There were 128 active UC patients and 144 in remission. We found that NLR of the active UC patients (2.89; IQR 2.2–4.26) were significantly higher than those in remission (1.78; IQR 1.38–2.44). The median PLR in patients with active and remission UC was 161 (IQR 116-216.6) and 121 (IQR 96.7-144.6), respectively (*P*<0.001）In the remission group, disease extent was proctitis in 45 patients (30%), left-sided in 56 (37.3%), and pancolitis in 49 patients (32.6%). Among active patients group, the majority (59.6%) were pancolitis.

We evaluated the association between disease extent and NLR, PLR. Disease extent was related to the NLR and PLR. Pancolitis showed the highest level of NLR (2.54; IQR 1.76–4.13), compared to left-sided colitis (2.47; IQR 1.61–3.44) and proctitis (1.75; IQR 1.37–2.6) (*P* < 0.01). Patients with pancolitis showed higher concentrations of PLR (141.93; IQR 102.7–216.7) than left-sided colitis (138.8; IQR 112.8–171.6) and proctitis (119.5; IQR 98.6–158.5) (*P* = 0.026).

### Correlation analysis of NLR and PLR with inflammatory markers

NLR was positively associated with CRP (*r =* 0.498, *p* < 0.01), ESR (*r =* 0.398, *p* < 0.01) and FC (*r =* 0.299, p < 0.01). A positive correlation was observed between PLR and CRP (*r =* 0.433, *p* < 0.01), ESR (*r =* 0.419, *p* < 0.01) and FC (*r =* 0.307, *p <* 0.01) (Table 3).

**Table 3:** Spearman correlation coefficients between NLR or PLR and other inflammatory markers in patients with UC

	NLR		PLR	
	r value	*P* value	r value	*P* value
ESR	0.398	<0.001	0.419	<0.001
CRP	0.498	<0.001	0.433	<0.001
FC	0.299	<0.001	0.307	<0.001

### ROC analysis

**Fig. 1:**
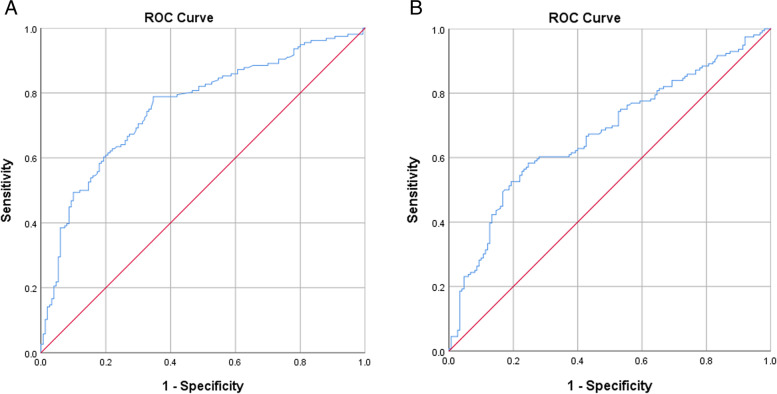
Receiver operating characteristic curves of NLR or PLR for differentiating active from inactive UC. **A**. ROC curve of NLR. **B**. ROC curve of PLR

We conducted the receiver-operating characteristic (ROC) curve analysis to determine specific cut-off values of biomarker for predicting activity in UC. The area under the curve (AUC) of NLR was 0.756 (95% CI 0.702 to 0.811) and the cut-off value was 2.19, with a sensitivity of 78.8% and specificity of 65%. AUC of PLR was 0.673 (95% CI 0.613 to 0.733) and the cut-off value was 147.96, with a sensitivity of 58.3% and specificity of 75% (Fig. 1). The cut-off value, sensitivity and specificity were also made for ESR, CRP and FC shown in Table 4.

**Table 4:** Accuracy for differentiating active from inactive UC using inflammatory markers

Variables	AUCs	SE	95% CI		Cut-offs	Sensitivity	Specificity
			Lower	Upper			
NLR	0.756	0.028	0.702	0.811	2.19	0.788	0.65
PLR	0.673	0.031	0.613	0.733	147.96	0.583	0.75
CRP	0.813	0.025	0.765	0.861	5.01	0.649	0.859
ESR	0.781	0.027	0.727	0.835	17.5	0.604	0.842
FC	0.822	0.028	0.77	0.877	653.5	0.754	0.778

*AUC* Area under curve, *CI* Confidence interval, *SE* Standard error, *ESR* Erythrocyte sedimentation rate, *CRP* C-reactive protein, *NLR* Neutrophil to lymphocyte ratio, *PLR* Platelet to lymphocyte ratio, *FC* Fecal calprotectin

## Discussion

In our retrospective study in a single UC cohort, we found that PLR and NLR were elevated in active UC patients in comparison to those in remission. NLR and PLR are positively correlated with inflammatory markers, such as CRP, ESR and FC. This is similar to previous studies. In a retrospective cohort including 119 active UC patients and 77 inactive patients, a NLR cut-off level of 2.16 indicated active UC [17]. In a Korean study, the cutoff value of NLR and PLR for detecting UC was 2.26 and 179.8, respectively [19]. A retrospective study compared the NLR of patients with UC during remission and active phase, and found the mean NLR of UC patients during active and inactive phase was 4.78 and 2.01. The ROC curve analysis revealed that the NLR and PLR cut-off level for active UC was 2.2 and 133.87, respectively [20]. A Turkish study of 71 UC patients and 140 controls indicated the cut-off value of NLR for active UC was 2.3 9 [21]. Another case–control study including 80 UC patients, NLR level in UC patients with active phase were higher than those in remission and controls. Using a ROC curve, NLR cut-off level of 1.9 predicted active UC [22]. NLR and PLR may be simple measures of disease severity in UC.

Neutrophils are the first leukocytes to be recruited to the inflammatory site with the capacity to kill pathogens. Besides their ability to eliminate pathogens, neutrophils take part in the immune response [23]. Interleukin-6 (IL-6), tumor necrosis factor-α (TN F-α) and other pro-inflammatory cytokines play a role in the activation and release of neutrophils. Neutrophil accumulation and crypt abscesses are classic features of inflamed mucosa of UC. Excessive accumulation of neutrophils in the intestine is associated with the disease severity and mucosal injury [24–26]. The absolute neutrophil count and the platelet count is frequently increased in IBD [27].

Thrombocytosis may occur in UC and indicate active disease. Platelet count is regarded as a useful measure of systemic inflammation. A positive correlation was observed between platelet counts and disease severity. Clinically active UC patients have a higher platelet count than those with inactive UC. It is also reported that platelet count is associated with disease relapse in UC [28–30]. Similarly, we observed that both neutrophil counts and platelet counts were elevated in the clinically active patients as compared to those in remission.

From this retrospective cohort study, we found PLR and NLR were positively correlated with FC. We also evaluated the cut-off value of FC to predict clinical remission. Previous study reported that the cut-off FC value of 200 μg/g indicated mucosal healing [31]. Our study found the cut-off FC level for clinical remission determined as Truelove and Witts was 653 μg/g. Lee et al also found a high cut-off FC value for clinical remission (1272.0 mg/kg), they defined the clinical remission by partial mayo score [32]. The sample size, study design and varying definitions of remission may lead to different results.

Our study has several limitations. A key limitation was the retrospective study design, we did not enroll all consecutive UC patients due to loss of laboratory examination data. The enrolled participants come from a single center with all Chinese subjects. Another limitation is that patients in the mild group based on Truelove and Witts criteria were classified as remission, we are unable to estimate endoscopic disease activity and histological remission in this study, because endoscopy had not been performed in some patients. We did not evaluate the impact of treatment, such as the use of immunomodulators, corticosteroids and biologic agents, which could potentially affect the leukocyte and thus the value of NLR and PLR [33]. Our study found male predominance in the active UC cases, the influence of gender on NLR and PLR were not investigated. Additionally, our study did not identify biomarkers that are clearly superior to ESR and CRP. However, NLR and PLR offer the advantage of being easily attainable and low cost in clinical practice. They are simple, easily measured and may be evaluated in every institution. Further studies are needed to determine whether combination of NLR, PLR and other non-invasive markers may be more useful predictors of disease activity. Finally, co-morbid conditions and nonspecific inflammation may influence inflammatory responses in patients with UC, we did not evaluate the association due to insufficient data.

In conclusion, our study indicated that NLR and PLR could be used in patients with UC as noninvasive markers of disease activity. Further study with prospective design and larger numbers of UC cases is warranted to explore the potential role of NLR and PLR in predicting disease activity.

## Data Availability

The data that support the findings of this study are available from the corresponding author upon reasonable request.

## Abbreviations

UC: Ulcerative colitis
PLR: Platelet-to-lymphocyte ratio
NLR: Neutrophil-to-lymphocyte ratio
CRP: C-reactive protein
ESR: Erythrocyte sedimentation rate
FC: Fecal calprotectin
IL-6: Interleukin-6
TN F-α: Tumor necrosis factor
5-ASA: 5-aminosalicylates
IQR: Inter-quartile range
AUC: Area under curve
CI: Confidence interval
SE: Standard error
ROC: Receiver-operating characteristic
